# People's willingness to pay for health insurance in rural Vietnam

**DOI:** 10.1186/1478-7547-6-16

**Published:** 2008-08-11

**Authors:** Curt Lofgren, Nguyen X Thanh, Nguyen TK Chuc, Anders Emmelin, Lars Lindholm

**Affiliations:** 1 Umeå International School of Public Health, Umeå University, Sweden; 2 Institute of Health Economics, Edmonton, Canada; 3 Dept. of Health Economics, Faculty of Public Health, Hanoi Medical University, Vietnam

## Abstract

**Background:**

The inequity caused by health financing in Vietnam, which mainly relies on out-of-pocket payments, has put pre-payment reform high on the political agenda. This paper reports on a study of the willingness to pay for health insurance among a rural population in northern Vietnam, exploring whether the Vietnamese are willing to pay enough to sufficiently finance a health insurance system.

**Methods:**

Using the Epidemiological Field Laboratory for Health Systems Research in the Bavi district (FilaBavi), 2070 households were randomly selected for the study. Existing FilaBavi interviewers were trained especially for this study. The interview questionnaire was developed through a pilot study followed by focus group discussions among interviewers. Determinants of households' willingness to pay were studied through interval regression by which problems such as zero answers, skewness, outliers and the heaping effect may be solved.

**Results:**

Households' average willingness to pay (WTP) is higher than their costs for public health care and self-treatment. For 70–80% of the respondents, average WTP is also sufficient to pay the lower range of premiums in existing health insurance programmes. However, the average WTP would only be sufficient to finance about half of total household public, as well as private, health care costs. Variables that reflect income, health care need, age and educational level were significant determinants of households' willingness to pay. Contrary to expectations, age was negatively related to willingness to pay.

**Conclusion:**

Since WTP is sufficient to cover household costs for public health care, it depends to what extent households would substitute private for public care and increase utilization as to whether WTP would also be sufficient enough to finance health insurance. This study highlights potential for public information schemes that may change the negative attitude towards health insurance, which this study has uncovered. A key task for policy makers is to win the trust of the population in relation to a health insurance system, particularly among the old and those with relatively low education.

## Background

Health financing in Vietnam relies mainly on out-of-pocket payments, which in 2000 were estimated to constitute as much as 80% of total health care expenditure [1]. More recent estimates are somewhat lower – around two-thirds [2]. The share of households facing catastrophic health care expenditure may be as high as 10% [3]. In this context, the need for furthering prepayment reform in Vietnam has been highlighted by many, and it is the goal of the Vietnamese government to achieve health insurance coverage for all citizens by 2010 [4].

Today there are two forms of health insurance for the Vietnamese: firstly compulsory health insurance for those that have formal employment, which was introduced in 1993 and now covers 9% of the population; secondly, there is voluntary health insurance, which was introduced in 1994 and now covers 11% of the population. In addition there are two programs: Health Care Funds for the Poor, which in 2003 replaced the Free Health Care Cards for the Poor, and free health care for children 0–5 years of age, which was established in 1991. Today these programs cover 18% and 11% of the population, respectively [2,5].

This means that around half of the population today is covered by health insurance or the two special programs. The task now is to attain coverage for the remaining half, which will, most likely, be a more difficult task [2,6].

This paper reports on a study of willingness to pay (WTP) for health insurance in Bavi, a rural district in northern Vietnam. Most of the inhabitants of Bavi are farmers who are not covered by health insurance. To our knowledge there is no other study of willingness to pay for health insurance in Vietnam, and few other studies of WTP for health care in the country; we found only one estimating WTP for obstetric delivery preferences [7]. There are, however, a number of other studies on health insurance in Vietnam, particularly on the effects on health care utilization and household out-of-pocket health expenditure. Several studies from recent years have found that voluntary health insurance is likely to increase considerably the visits to health care facilities and reduce out-of-pocket spending [8-10], whilst also leading to less self-treatment (buying of drugs without medical advice from professionals) [11,12]. Compulsory insurance has been found to increase health care utilization more than voluntary health insurance [13], and the Health Care Fund for the Poor also appears to increase the use of health services, particularly inpatient care [5]. These findings are of interest for our study, especially concerning the question of whether the WTP we have estimated is sufficient to finance viable health insurance. This is discussed below in relation to our results.

WTP for health insurance has been studied in other developing countries, although the number of studies is relatively small. In a study from a city in China, the WTP of informal sector workers to join an existing health insurance package for formal workers has been studied [14]. The average WTP was found to be higher than the cost of expanding such an insurance system. In Burkino Faso, the feasibility of a community-based health insurance package was studied in a rural area. Based on the WTP estimates, it was found to be feasible if health service utilization did not increase by more than 28% [15,16]. In Ghana a WTP study of informal sector workers showed that 64% would sign up for health insurance for a reasonable (compared to costs) premium [17]. In Iran it was found, based on the respondents' WTP, that the existing health insurance system in urban areas could be introduced in rural areas [18], and finally, a WTP study in a rural area in India was used as a basis for discussing the content of health policy reform [19]. In the absence of WTP studies of health insurance in Vietnam, the above studies from other countries are of interest as reference points for our findings on the determinants of WTP. These comparisons are made in the discussion section.

We first present the methods used, including the rationale for using the WTP technique, the study design, the surveillance system used to collect the data, hypotheses about determinants for WTP and the method used to elicit WTP. This is followed by discussion of the econometric method used; due to the typical heaping of WTP answers we have used interval regression. Results are then presented and finally a methodological discussion, including potential bias, and a discussion of the results and their policy implications.

## Methods

It is becoming increasingly popular in health economics to use the WTP approach to elicit the value people place on health and health care activities [20]. In the absence of monetary measurements of such values found on functioning markets – where consumers reveal how much of other goods they are willing to sacrifice to get a certain product – researchers instead ask potential consumers how much they would be willing to pay [21]. An advantage of this technique is that it measures the strength of consumer demand in monetary units, which can then be compared to costs [22]. Respondents are presented with a hypothetical scenario and then asked about their maximum willingness to pay for, for example, joining a health insurance scheme. Below we present the basis for data collection, followed by the design of our WTP study.

In 1999, in collaboration with Vietnamese and Swedish public health scientists, the Epidemiological Field Laboratory for Health Systems Research (FilaBavi) was established in the Bavi district of Vietnam, whose centre lies some 60 km west of Hanoi [23]. In 1999 a baseline household survey was undertaken followed by quarterly surveillance of vital events and complete re-surveys every two years.

The Bavi district has a population of 235,000. For the surveillance database a random selection of 67 out of 352 clusters was made, with probability proportional to size. This means that we do not have to adjust for clustering effects in the estimations.

The surveillance database includes a population of 51,024 in 11,089 households. Each cluster was based on a village and consisted of 41 to 512 (mean 146) households with a population of 185 to 1,944 (mean 676). The largest clusters were then divided into 3, thereby in total there are 69 clusters in FilaBavi.

In 2004, 30 households were randomly selected for the present study from each cluster in the FilaBavi surveillance database, which gives a total of 2,070 households. Of these, complete interviews were held within 2,063 households. The aim of this study was to interview the heads of households only, most of which are men. In the FilaBavi database this share is 62%. To ensure that there would be a reasonable proportion of female respondents, households were deliberately selected for this study so that half of the household heads would be women.

To interview only heads of households, however, turned out to be too time consuming. Therefore, interviewers restricted themselves to interviewing the head of the household if this person was at home at the time of the interview, or the spouse if the head could not be contacted; in total, 51% of the respondents were heads of households (table 1). An indicator variable has been included in the regression models to control for possible bias in relation to this. Of the interviewed household heads 44% were female, but of the total number of interviewees 64% were female. There is an indicator variable in the estimations controlling for gender. However, it should be recognized that there is a validity problem concerning the selection of households since female-headed households may be more disadvantaged than others. This is analyzed in the discussion section.

**Table 1:** Respondent and household characteristics

Variable name	Description	Mean*	Std.dev
Male	Male = 1, female = 0	0.36	
Age	Age in years	44.57	13.58
Farmer	Farmer = 1, all other occupations = 0	0.74	
Morethanprimary	More than primary education = 1, otherwise 0	0.70	
Membershh	Number of members in the household	4.01	1.56
Children	Number of children, 0 to 5 years age, in the household	0.37	0.64
Elderly	Number of persons, 65 years and older, in the household	0.32	0.58
Chronic	One or more persons in the household has a chronic disease = 1, 0 otherwise	0.20	
Hcneed	At least one person in the household needed health care during the last year = 1, 0 otherwise	0.92	
Insureexp	The household has insurance (of any kind) = 1, 0 otherwise	0.18	
Poor	The household is classified as poor by local leaders = 1, 0 otherwise	0.11	
Rich	The household is classified as rich by local leaders = 1, 0 otherwise	0.16	
Head	The respondent is the household head = 1, 0 otherwise	0.51	

*The mean value for indicator variables shows the proportion for the category which assumes the value 1. For e.g. the variable Farmer, the mean value shows that 74% of the respondents are farmers.

This is a study of household WTP, rather than individual WTP, as the economic decision to purchase health care among these rural and mostly farmer households is more likely to be a household decision and not an individual one. This is a common approach when studying rural communities in developing countries. Of the six previously cited studies of WTP for health insurance in developing countries (other than Vietnam), four of them estimate household WTP.

The interviewers in this study conduct regular surveys for the FilaBavi database. They are all educated to at least high school level and have received special training for their task. For testing the questionnaire, in particular the scenarios, a pilot of 15 in-depth interviews with heads of households outside the study sample was performed by the researchers. The version of the questionnaire developed on that basis was then discussed in four focus groups consisting of interviewers. The purpose of the focus groups was for training of the interviewers and further refining of the questionnaire. Before going to the field, the interviewers were trained twice, using a role-play technique on how to use the questionnaires. They were strictly supervised throughout the study period.

**Figure 1:**
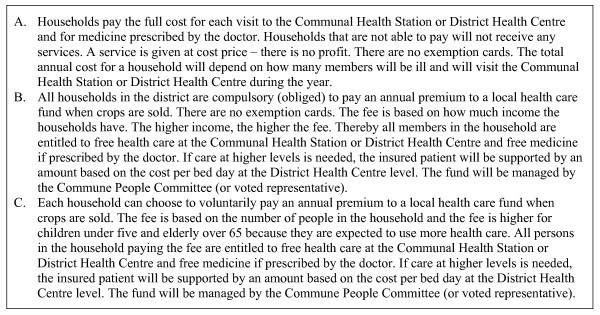
Hypothetical scenarios.

The choice set described and explained to respondents is presented in Figure 1. It consists of three different health care financing systems: A was an out-of-pocket model similar to the present system in Bavi, whilst B and C had identical benefit packages but were based on different financing schemes. B was a compulsory health insurance scheme based on community rating, and C was a voluntary scheme based on risk rating.

The three alternatives cover different financing systems for public health care, which is obvious from the scenarios but was also clearly pointed out to respondents. The respondents were asked to choose which one of these health financing systems they would prefer to have in Bavi. All respondents (not only those that preferred B or C respectively) were then also asked about their WTP for system B, given that this system would be implemented in Bavi, and similarly for system C, given that system C would be implemented. The WTP question was of a Yes/No nature in relation to a certain bid (insurance cost), with a follow-up question about maximum WTP.

The bid was calculated based on another study from FilaBavi [24] where the average health care costs for households within the district was estimated (table 2); in 2002 this was 520,000 VND per year, which corresponds approximately to 45,000 VND per month. This later figure was used as the bid given to respondents, who were asked: Given that system B/C is chosen, would you be willing to pay 45,000 VND per month for your household? Respondents were then given an open question about their maximum WTP in each system. The WTP elicited using the above method is presented in the results section.

**Table 2:** Average household expenditure for health care in Bavi, July 2001 to June 2002, Vietnamese dong

	for the whole year	%	average per month
**Public health care**	129 267	25%	10 772
Commune health stations	23 698	5%	1 975
District health centres	45 621	9%	3 802
Provincial hospitals	32 508	6%	2 709
Central hospitals	26 895	5%	2 241
Others	545	0%	45
**Private health care**	283 342	55%	23 612
**Self-treatment**	60 338	12%	5 028
**Total curative exp**	472 947	91%	39 412
Health insurance	16 227	3%	1 352
Prevention & rehabilitation	29 317	6%	2 443

**Total**	518 491	100%	43 208

*Source: *Thuan NTB: **The burden of household health care expenditure in a rural district in Vietnam**. *MPH thesis*. Nordic School of Public Health, Sweden; 2002

In the scenarios nothing was said about the respondents' expected health-seeking behavior. According to table 2, it is clear that public health care stands for less than half of total health care expenditure in Bavi. A very large share for private health care was also found in a nationwide study using the Vietnam Living Standard Survey 97/98 [25]. In the background section above studies on the effects of health insurance in Vietnam were cited. It appears that one can expect that a growing number of persons signing up for health insurance will lead to increased utilization of public health services and less self-medication – a shift away from private to public services.

However, when presenting respondents with a WTP scenario it is very important that it can be clearly understood. We concluded that complicating the scenario by adding information about an expected change in health-seeking behavior would make it too complex. But this of course leads to uncertainty when interpreting the elicited WTP, a question addressed in the discussion section below.

In relation to this we based the bid to the respondents on the total (public as well as private) household health care expenditure. This includes not only curative expenditure but also expenditure for health insurance (3%) and for prevention and rehabilitation (6%) (table 2). The curative expenditure includes costs for consultations, drugs and tests and for traveling (6%) and lodging (2%) (unpublished data from [24]). We wanted households to consider WTP based on total health care costs although we did not specify or point to a possible substitution of providers.

Our choice of background variables (see table 1), which were also collected through the interviews, follow our hypotheses about the determinants for WTP. Health insurance demand is a function of, apart from the price of the insurance, the respondent's degree of risk aversion, perceived risk of injury/illness, perceived extent of the loss caused by illness/injury, and income [26].

Using insurance theory, assuming a decreasing marginal utility of income, it follows that the higher the degree of risk aversion, the higher WTP will be when all else is equal. This is also the case for the perceived extent of the loss incurred by illness or injury. For the perceived risk of illness or injury, however, the relationship is not this simple; for a small – and a large – risk, WTP may be relatively small. If the risk is 1, illness will occur with certainty, and the individual is better-off not buying insurance (including a load factor) with a risk-rated premium. If the insurance is based on community rating, this individual may still benefit from insurance, however. We assume that the risks perceived by the households in this study are not in the relatively large risk segment, so that it is reasonable to hypothesize that an increase in perceived risk, all else being equal, leads to an increase in WTP. We also hypothesize that the higher the income, the higher the WTP.

**Figure 2:**
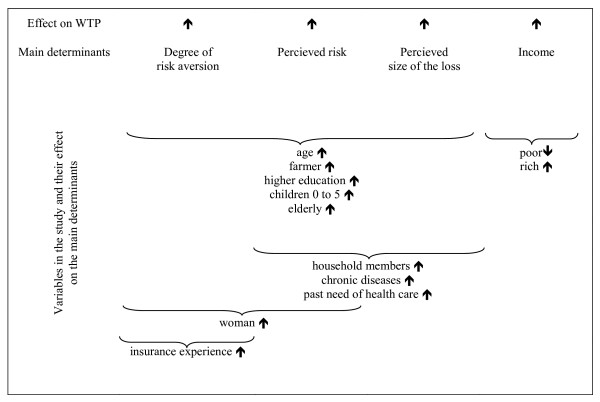
The main determinants of WTP and the variables.

Figure 2 illustrates the hypothesized effects of the study variables on the main determinants of WTP.

We hypothesize that five variables will affect risk aversion, the perceived extent of the loss and the perceived risk amongst respondents, namely; age, occupation, educational level, and the number of children and elderly in the household. The older the respondent is, the higher the perceived risk will be for him/her. We assume that the degree of risk aversion increases with age, as does the perceived extent of the loss. An older person has more experience and can therefore more accurately envisage the affect of illness or injury on their household.

Farmers may be more vulnerable than other occupational groups, as illness/injury during critical periods of the year, such as at harvest, may have a proportionally greater affect on income than the duration of illness/injury. We can assume that respondents who have been educated to a relatively high level will have more knowledge about the effects of and need for health care due to illness. Finally, risk is also higher for children and the elderly, therefore risk aversion, perceived loss and risk may be higher the more children and elderly there are in a household.

The total number of household members and the number amongst them with chronic diseases are assumed to increase the perceived extent of the loss, as well as the perceived risk. Utilization of health care during the last year may also be an indicator of greater awareness of what might happen in case of illness/injury.

We employ the common assumption that women have a higher degree of risk aversion than men and that they have a higher risk of illness. Finally, households that have some sort of insurance (not only health insurance) have shown that they have a greater risk aversion than those with no insurance.

We have discussed above individual (or household) determinants of WTP. An interesting discussion today concerns the importance of "social determinants" in the form of social capital that could significantly affect household preferences for health insurance [27]. There is no clear consensus surrounding the definition of social capital [28], but it is generally agreed that it concerns informal networks that are established between households, and furthermore the trust and solidarity that characterizes these networks [27].

Interestingly, the existence of social capital may affect WTP for health insurance both positively and negatively. To the degree that households trust one another in a community, they may also trust community-based health insurance schemes similar to those presented in the scenarios, which would, all else being equal, increase WTP. However, the existence of informal risk-sharing networks may also tend to "crowd out" formal health insurance, which would lead to lower WTP [27,28]. Unfortunately we have no information about and no variables that measure social capital, the implications of which are explored in the discussion section below.

There are four problems common to many WTP studies: i) the distribution of stated WTP is skewed; ii) some respondents will state a zero WTP; iii) other respondents will state a WTP very different from most of the respondents (outliers); and iv) respondents' WTP will tend to concentrate – "heap" – around certain values.

Skewness is often dealt with by using a log-normal model. The zero cases will then have to be excluded and outliers are also often excluded based on different criterions. The heaping effect, however, is often ignored. The fact that respondents appear to concentrate on convenient values suggests that their stated WTP represents a certain interval, rather than a precise amount. Torelli and Trivelato [29] have shown that this behaviour, if not considered, may disguise true relationships.

The heaping effect in our data is illustrated in table 3. About one-fifth of the respondents state a zero WTP in system B and almost one-third do so in system C. It is obvious from table 3 that the other respondents concentrate on values such as 5,000, 10,000, 15,000 VND and so on. It is also noteworthy in table 3 that one respondent stated a WTP of 22 VND, which is an amount that hardly differs from zero in this context. This is addressed further in the methodological part of the discussion section.

**Table 3:** Household WTP in the two insurance systems

Compulsory insurance (B)			Voluntary insurance (C)		
Stated WTP	No of households	Percent	Stated WTP	No of households	Percent

0	438	21%	0	617	30%
22	1	0%	2 000	5	0%
2 000	4	0%	3 000	6	0%
3 000	10	0%	4 000	1	0%
4 000	1	0%	4 500	1	0%
4 500	1	0%	5 000	115	6%
5 000	120	6%	7 000	1	0%
7 000	3	0%	7 500	1	0%
8 000	2	0%	8 000	2	0%
10 000	378	18%	10 000	334	16%
15 000	158	8%	12 000	1	0%
18 000	1	0%	15 000	141	7%
20 000	453	22%	20 000	395	19%
22 000	4	0%	22 000	3	0%
22 500	4	0%	22 500	7	0%
25 000	40	2%	25 000	41	2%
27 500	1	0%	27 500	2	0%
30 000	112	5%	30 000	105	5%
35 000	5	0%	35 000	2	0%
40 000	7	0%	40 000	7	0%
45 000	261	13%	45 000	223	11%
50 000	36	2%	50 000	35	2%
60 000	4	0%	55 000	1	0%
70 000	3	0%	60 000	5	0%
80 000	1	0%	70 000	3	0%
90 000	1	0%	80 000	1	0%
100 000	10	0%	90 000	1	0%
150 000	1	0%	100 000	6	0%
200 000	2	0%	225 000	1	0%
225 000	1	0%			

Total	2 063	100%	Total	2 063	100%

If we assume that respondents' stated WTP represents intervals rather than precise measurements then this must be considered in the econometric method. We have done so by using interval or grouped data regression [30]. We estimate the following model:

Suppose $y_{i}^{*}$ represents respondents' true WTP, which is a variable we cannot observe. What we do observe is another variable *y*_*i *_for which

*y*_*i *_= 1 when $y_{i}^{*}$ ≤ 2 500 VND

*y*_*i *_= 2 when 2 500 <$y_{i}^{*}$ ≤ 7 500 VND

*y*_*i *_= 3 when 7 500 <$y_{i}^{*}$ ≤ 12 500 VND

*y*_*i *_= 4 when 12 500 <$y_{i}^{*}$ ≤ 17 500 VND

*y*_*i *_= 5 when 17 500 <$y_{i}^{*}$ ≤ 22 500 VND

*y*_*i *_= 6 when 22 500 <$y_{i}^{*}$ ≤ 27 500 VND

*y*_*i *_= 7 when 27 500 <$y_{i}^{*}$ ≤ 32 500 VND

*y*_*i *_= 8 when 32 500 <$y_{i}^{*}$ ≤ 37 500 VND

*y*_*i *_= 9 when 37 500 <$y_{i}^{*}$ ≤ 42 500 VND

*y*_*i *_= 10 when 42 500 <$y_{i}^{*}$ ≤ 47 500 VND

*y*_*i *_= 11 when 47 500 <$y_{i}^{*}$ ≤ 52 500 VND

*y*_*i *_= 13 when 52 500 <$y_{i}^{*}$

Suppose that

ln $y_{i}^{*}$ = *βx*_*i *_+ *ε*_*i *_where *ε*_*i *_~ N(0, *σ*^2^)

In this case the likelihood function is

$$\begin{array}{l} L=\prod_{y_{i}=1}\Phi \left(\frac{\ln2500-\beta x_{i}}{\sigma }\right)*\\ {}*\prod_{y_{i}=2}\left[\Phi \left(\frac{\ln7500-\beta x_{i}}{\sigma }\right)-\Phi \left(\frac{\ln2500-\beta x_{i}}{\sigma }\right)\right]*\\ {}*\prod_{y_{i}=3}\left[\Phi \left(\frac{\ln12500-\beta x_{i}}{\sigma }\right)-\Phi \left(\frac{\ln7500-\beta x_{i}}{\sigma }\right)\right]*•••\\ \begin{array}{cc} ••• & *\prod_{y_{i}=11}\left[\Phi \left(\frac{\ln52500-\beta x_{i}}{\sigma }\right)-\Phi \left(\frac{\ln47500-\beta x_{i}}{\sigma }\right)\right]* \end{array}\\ {}*\prod_{y_{i}=12}\left[1-\Phi \left(\frac{\ln52500-\beta x_{i}}{\sigma }\right)\right] \end{array}$$

Using interval or grouped data regression solves the problems mentioned above and the heaping effect is considered. Also, the logarithm of the dependent variable can be used adjusting for skewness. Still, zero answers for WTP can be included. (If someone imagines the existence of negative WTP reflected in zero answers this is also included.) Outliers are kept in the highest interval.

The likelihood function has been maximized using STATA 8.0.

The Research Ethics Committee at Umeå University has given ethical approval for the FilaBavi household surveillance system, including data collection on vital statistics (reference number 02-420), and specific approval for the stated preferences survey (§86/04). The study has also received ethical approval from Hanoi Medical University and the Ministry of Health in Hanoi. The interviewers obtained informed consent for the interviews from heads of households.

## Results

In the choice between the three different financing systems presented in Figure 1, a majority (52%) of respondents preferred out-of-pocket financing, system A. Among the rest, preferences were stronger for compulsory (28%) rather than voluntary (20%) health insurance. The results of the choice experiment are reported in Thanh et al. [31], where the determinants for the choice between the three systems are also studied.

The focus of the present paper is on the extent and determinants of WTP for health insurance. The respondents were asked two different types of questions; the first – analyzed in Thanh et al. [31] – concerned the choice of financing system and aimed to explore which of the three systems the respondents prefer over the others; in the second type of question – analyzed in this paper – respondents were asked how much they would be willing to pay ***given ***that a certain system (B or C) was chosen for Bavi. All of the respondents were asked these WTP questions, and not only those who preferred insurance over out-of-pocket. Below we first report the extent of WTP given the respective systems, and then present the estimations of what determines WTP.

The average household in Bavi spends about 520 000 VND per year or around 45 000 dong per month for health care of all sorts – private as well as public with both curative and preventive care. This finding is from a study within the FilaBavi project and was used as the starting bid in this study (table 2).

The average household WTP is lower than this, however (table 4). *For the compulsory insurance *the average household WTP is around 18 000 dong per month. *For the voluntary insurance *it is even lower. If only those respondents who have a positive WTP are included, or only those households that prefer one of the health insurance alternatives over out-of-pocket financing, the average is 22 000–24 000 VND in the respective schemes. This elicited WTP corresponds to half of the total health care expenditure of the average household in Bavi.

**Table 4:** Respondents' WTP for the two forms of health insurance

	For household per month		Per person and year*			
	Mean	Median	Mean	Median	% of respon- dents	N

**Compulsory health insurance**						
WTP for all respondents	17 873	15 000	47 661	40 000	100%	2 063
WTP for respondents whose WTP > 0	22 690	20 000	60 507	53 333	79%	1 625
WTP for respondents who prefer HI over OOP	23 650	20 000	63 067	53 333	48%	999
						
**Voluntary health insurance**						
WTP for all respondents	15 588	10 000	41 568	26 667	100%	2 063
WTP for respondents whose WTP > 0	22 239	20 000	59 304	53 333	70%	1 446
WTP for respondents who prefer HI over OOP	22 501	20 000	60 003	53 333	48%	999

*Average household size is 4.5 persons.

HI = health insurance.

OOP out-of-pocket payments

Total household health expenditure covers public health care (11 000 VND), self-treatment (5 000 VND) and private health care (24 000 VND), which gives a total of 40 000 VND (table 2). Added to this is the cost of health insurance, prevention and rehabilitation, which gives a total of around 45 000 VND, hence the starting bid for respondents. Thus, the average WTP for all respondents covers more than the costs for public health care and self-treatment but does not cover costs for private care. Whether one should conclude that this represents a favourable basis for the expansion of health insurance in this district depends, among several things, on the assumptions one makes about how respondents are likely to behave once insured – to what extent would they substitute self-treatment and private health care for public health care, and to what extent would they increase their demand for health care? This is discussed in the next section. As a basis for the discussion we will below compare to existing insurance premiums.

Health insurance systems operate in Vietnam where the premiums correspond to a lower level of household health care expenditure than reported above for Bavi. For the community-based health insurance schemes offered in rural areas by the Vietnam Social Security, premiums range from 60,000 VND to 100,000 VND per person and year. [32]. The lower boundary of this range corresponds to 22 000 VND per household and month in Bavi, i.e. an amount equal to the WTP of households whose WTP is larger than zero. These groups of households make up 70% (for the voluntary insurance system) and 80% (for the compulsory insurance system) of the total group of households (table 4). The Vietnam Social Security also offers a school health insurance system for students [33], for which premiums range from 10,000 VND to 45,000 VND per student and year. The upper boundary of that range is close to the average WTP for all households in this study.

We have compared a low-cost health care system to the income that would be generated through the WTP stated by the respondents. This is done for those in the Bavi population who prefer health insurance (compulsory or voluntary) over out-of-pocket health care payments. The estimation is explained in more detail in appendix 1. We assume that the uninsured population who prefer health insurance, enrol in a health insurance scheme. We also assume that their health care utilization matches the national average and that non-treatment and self-treatment episodes are replaced by outpatient care at Community Health Centres. Furthermore, we assume that private users turn to public health care with the same patterns as public users. Finally, we assume that the length of stay at the provincial and central levels is the same as at the district level (see the WTP scenarios in Figure 1).

The total health care costs incurred by the target population per year were estimated as being 5.9 billion VND. The stated WTP for the same population would yield an income of the same magnitude, ranging from 5.6 to 5.9 million VND (table 5) based on a WTP between 60,000 and 63,000 VND per person per year.

**Table 5:** Total yearly income for a health insurance scheme and estimated health care costs

Health insurance scheme	WTP per household and month (1)	Household members (2)	Premium per person and month (3)	Premium per person and year (4)	Enrolees (5)	Total yearly income (6)
Compulsory (B)	23,650	4.5	5,256	63,067	93,949	5,925,050,266
Voluntary (C)	22,501	4.5	5,000	60,003	93,949	5,637,190,530

	Health care costs per household and month (12)	Household members (11)	Health care costs per person and month (10)	Health care costs per person and year (9)	Enrolees (8)	Total health care costs (VND) (7)

	23,572	4.5	5,238	62,858	93,949	5,905,491,555

Note: The health insurance schemes include only those households that prefer health insurance to out-of-pocket payments. For the estimation of health care costs see appendix 1.

(3) = (1)/(2).

(4) = (3)*12 months.

(6) = (4)*(5).

(9) = (7)/(8) [(7) is from table A1].

(10) = (9)/12 months.

(12) = (10)*(11)

The estimations of what determines WTP are presented in tables 6 and 7. As hypothesized, the income variables are significant determinants for WTP in system B and close to significant (or significant at the 10% level) in system C. Being a rich household is significant, or close to significant, and positive in some of the estimations. Belonging to the group of poor households is significant, or close to significant, and negative in some of the estimations.

**Table 6:** Interval regression. WTP determinants for compulsory health insurance (system B)

	1			2		
	Coef.	z	P > |z|	Coef.	z	P > |z|

Head	.0621513	0.87	0.382	.042933	0.66	0.507
Male	.0785897	1.12	0.261	.029905	0.47	0.638
Age	-.0106958	-3.83	0.000	-.0102374	-4.03	0.000
Farmer	-.0367228	-0.51	0.613	-.1386146	-2.09	0.036
Morethanprimary	.1528767	2.24	0.025	.1391636	2.25	0.025
Membershh	.1107999	5.35	0.000	.0826442	4.38	0.000
Children	.0003272	0.01	0.995	-.0011396	-0.03	0.979
Elderly	-.0356463	-0.65	0.518	-.0058438	-0.12	0.907
Chronic	.1734691	2.43	0.015	.062738	0.97	0.335
Hcneed	-.1701406	-1.67	0.095	-.0932201	-1.00	0.315
Insurexp	.0495472	0.61	0.540	-.0144759	-0.20	0.844
Poor	-.1165337	-1.28	0.201	-.184308	-2.22	0.027
Rich	.1986089	2.45	0.014	.1885194	2.56	0.010
Prefcohi				1.129829	18.86	0.000
Prefvohi				.8976605	13.31	0.000
Constant	9.227955	47.50	0.000	8.89968	50.17	0.000
	Log likelihood = -5003.2461			Log likelihood = -4809.0038		
	LR chi2(13) = 111.20			LR chi2(15) = 499.68		
	Prob > chi2 = 0.0000			Prob > chi2 = 0.0000		

	Total number of observations = 2022			Total number of observations = 2022		

Variable name	Description					
Prefcohi	Prefcohi = 1 if the respondent prefers compulsory health insurance (system B) over the other alternatives. Prefcohi = 0 otherwise.					
Prefvohi	Prefvohi = 1 if the respondent prefers voluntary health insurance (system C) over the other alternatives. Prefvohi = 0 otherwise					

For an explanation of the other variables, see table 1.

**Table 7:** Interval regression. WTP determinants for voluntary health insurance (system C)

	1			2		
	Coef.	z	P > |z|	Coef.	z	P > |z|

Head	.1240847	1.47	0.142	.0740998	1.02	0.309
Male	.1294685	1.56	0.119	.0749992	1.05	0.294
Age	-.0083357	-2.52	0.012	-.007418	-2.60	0.009
Farmer	.0144706	0.17	0.867	-.112466	-1.51	0.131
Morethanprimary	.20443	2.52	0.012	.1703909	2.44	0.015
Membershh	.0996013	4.05	0.000	.0646929	3.05	0.002
Children	.1119569	1.97	0.048	.0792814	1.62	0.104
Elderly	-.0344205	-0.53	0.599	-.0092381	-0.16	0.870
Chronic	.3078385	3.65	0.000	.1541756	2.12	0.034
Hcneed	-.4161749	-3.47	0.001	-.3366622	-3.27	0.001
Insurexp	.0769168	0.80	0.423	-.0151158	-0.18	0.855
Poor	-.0419754	-0.39	0.697	-.153246	-1.65	0.100
Rich	.1770999	1.84	0.066	.1259169	1.52	0.129
Prefcohi				1.321762	19.56	0.000
Prefvohi				1.648773	21.81	0.000
Constant	8.885455	38.61	0.000	8.452932	42.49	0.000
	Log likelihood = -4820.0345			Log likelihood = -4522.9879		
	LR chi2(13) = 97.41			LR chi2(15) = 691.51		
	Prob > chi2 = 0.0000			Prob > chi2 = 0.0000		

	Total number of observations = 2022			Total number of observations = 2022		

Variable name	Description					
Prefcohi	Prefcohi = 1 if the respondent prefers compulsory health insurance (system B) over the other alternatives. Prefcohi = 0 otherwise.					
Prefvohi	Prefvohi = 1 if the respondent prefers voluntary health insurance (system C) over the other alternatives. Prefvohi = 0 otherwise					

For an explanation of the other variables, see table 1.

The larger the household the bigger the WTP. This holds true for all estimations. In system C, WTP is also higher as the number of children in the household increases. WTP is also higher for households that have at least one member with a chronic disease, and is true for three of the estimations. All of the estimations show that WTP is higher if the respondent is educated beyond primary level.

All of the above results were expected and are in line with our hypotheses. We did not expect, however, that WTP would fall with increasing age of the respondent, and that having at least one person in the household who needed health care during the last year would decrease WTP in three of the estimations. Also, being a farmer is significant and negative in one of the estimations.

## Discussion

### Methodological considerations

There are a large number of potential biases in a WTP study. We follow the typology developed by Mitchell and Carson [34] when discussing the biases relevant to our study and whether they may pose a problem or not. Mitchell and Carson classify the ("potential response effect") biases into three large groups:

i) The first group concerns cases where respondents misrepresent their true WTP. For example, this could be a *strategic bias *when a respondent purposely states a WTP higher or lower than the true one because the respondent in his or hers self-interest wants to influence the result of the study. It could also be a *compliance bias *when a respondent gives an answer he or she believes the interviewer wants to hear.

ii) The second group concerns cases where the elicitation method implicitly gives a "correct" value for the WTP. The *starting point bias *is one of these biases. A bid is given to the respondent and thereby a cue to where the WTP might lay.

iii) The third group concerns different misspecifications of the scenario. In this case the respondent perceives the scenario differently to what is intended. Among these biases, the *part-whole bias *is of particular interest to our study It means that the respondent includes something which is not in the scenario or excludes something which is there.

In our study, instead of choosing a direct open-ended WTP question (simply asking the respondent what his/her maximum WTP is) we chose a take it or leave it question with an open ended follow-up; the reason being that respondents may find it hard to answer direct open-ended questions and that this in turn may lead to many protest zero answers. With our format, there is instead a risk for a starting point bias, however, the results do not indicate that this is a problem. Most respondents give a WTP far lower than the bid they were given. Only 15% (for compulsory health insurance) and 13% (voluntary health insurance) stated a WTP equal to or higher than the bid they were given (table 3). The average WTP was less than half of the bid.

Some respondents did give a WTP equal to zero, 21% for the compulsory insurance and 30% for the voluntary insurance. But it is not likely that these were protest zeros in the sense discussed above. The scenario was carefully explained by the interviewers and a concrete bid was given. The interview process was closely monitored and the interviewers did not report any problems in making the bid understandable for the respondents. However, there could be WTP zeros given, not representing true WTP, for another reason; there may be a strategic bias. Almost all of the respondents (90%) stating a zero WTP belong to the group preferring the out-of-pocket financing alternative over the health insurance alternatives (tables 8 and 9).

**Table 8:** The number of respondents stating a zero WTP for the compulsory health insurance system

	Preference for financing systems			Total
	Out-of- pocket	Compulsory health insurance	Voluntary health insurance	

WTP = 0	394	5	39	438
	90%	1%	9%	100%
WTP > 0	671	582	372	1625
	41%	36%	23%	100%

Total	1065	587	411	2063
	52%	28%	20%	100%

**Table 9:** The number of respondents stating a zero WTP for the voluntary health insurance system

	Preference for financing systems			Total
	Out-of- pocket	Compulsory health insurance	Voluntary health insurance	

WTP = 0	557	59	1	617
	90%	10%	0%	100%
WTP > 0	508	528	410	1446
	35%	37%	28%	100%

Total	1065	587	411	2063
	52%	28%	20%	100%

For an explanation of the other variables, see table 1.

It may well be that some of them voted once more for their preferred system when they stated their WTP, even though the question was about their WTP given that someone else (the government) had chosen to implement a health insurance system. This may also be the case for the respondent who stated a WTP of 22 VND for compulsory health insurance, since this amount is very low indeed (table 3). We cannot determine to what extent this is a problem in our study. It was pointed out in the data section above that it is reasonable to assume that respondents have a larger (true) WTP for the financing alternative that they prefer, or conversely a lower WTP for the alternatives that they do not prefer. But if there is a strategic bias, WTP in this study is underestimated since there is no indication of inflated WTP answers (WTP being far lower than actual health care expenditure).

A compliance bias seems less likely because of the relatively low WTP given in relation to the bid. If the respondents wanted to please the interviewers they may be expected to give a WTP closer to the bid.

Another problem is found in the third group of potential biases described above; did the respondents understand the scenarios? Again, the interview process was well planned (including interviewer training and focus group discussions) and carefully monitored. There is therefore no reason to suspect that the respondents didn't understand the scenarios, however, they may not have trusted them.

The respondents may have generalized the problems of the existing health insurance systems in Vietnam to the hypothetical ones [31]. In reality, when using insurance, patients can risk longer waiting times and lower quality of care. They also run the risk of still having to pay considerable amounts out-of-pocket, e.g. in the form of gifts to the staff [9]. With this in mind, the respondents may not have believed or trusted that the health insurance described in the scenarios would deliver the benefits promised. If so there is an information bias. The WTP that respondents indicated may relate to benefits that are smaller than the intended benefits in the scenarios, and therefore the WTP may be underestimated.

The conclusion from this discussion of potential biases is therefore that there is a possibility that WTP estimates are underestimated for two reasons, strategic behavior and part-whole bias. The starting point and the compliance bias seem less likely.

It is also possible that WTP is underestimated in relation to the true WTP of the Bavi population, since the selection of households was conducted so that 50% of them would be headed by females. There is evidence that female-headed households are more disadvantaged than others [35,36]; that a larger percent of them live in poverty than other households. Since income is positively related to WTP, this could mean that households in this study have a lower WTP than those of the entire Bavi population.

### WTP for health insurance

The determinants of WTP in this study are mostly in line with our expectations; having a greater income, higher education, larger household and at least one household member with chronic disease increases WTP. We have not found any other WTP study of health insurance from Vietnam for comparison, but the results reported from WTP studies in other developing countries (cited in the background section) show similar results in these respects.

We did not expect WTP in the present study to fall with increasing age, and also if the household had been in need of health care during the last year. In the studies from other developing countries the results on age are mixed, some report increasing, and others decreasing WTP with age. When variables similar to our "hcneed"(if the household had been in need of health care during the last year) are included in studies from other countries, the result is opposite to ours, which is noteworthy and discussed below.

For WTP for health insurance our results can be summarized as follows:

• The average WTP (18 000 VND) covers the average costs for public health care (11 000 VND).

• The average WTP is also sufficient to include self-treatment (5 000 VND).

• For 70–80% of the respondents the average WTP (22 000) is sufficient to pay the lower range of premiums in the existing health insurance programme.

• It is feasible to design a low-cost health care system that could be financed – at least for the population who prefer insurance over an out-of-pocket system -given the WTP stated by the respondents.

• The average WTP would only be sufficient to finance about half of all health care costs, public as well as private.

The respondents were asked about their WTP for two insurance systems for public health care. These insurance systems would give them free health care and free prescribed drugs at the commune and district levels, and reimbursement at higher levels corresponding to the cost at the district level. In this situation there are two extreme alternatives for how the respondents could behave if insured:

1. They could substitute all private care for public care. Their WTP would not be sufficient to finance this.

2. They could continue using public health care at the same frequency as before. Their WTP would be enough to finance this.

Existing evidence indicates that something in between these two alternatives would happen. The studies on health insurance in Vietnam referred to in the background section show there will most likely be a shift from private care and self-treatment to public care, and that health service utilisation will increase. If these changes are substantial, the limit for what average WTP in this study can finance is soon reached.

There is a logical question here: In the situation these households are experiencing, with high out-of-pocket medical expenses and risk for catastrophic health expenditure, why do they not state a higher WTP? In the section above the possibility that WTP is underestimated was discussed. This is due both to a possible strategic and a part-whole bias. Some of the respondents who preferred out-of-pocket financing to insurance may have stated a zero WTP for insurance. Some respondents may also have interpreted things in the insurance scenarios that were not meant to be there.

One such factor may be the informal payments, in the form of money or gifts to the staff, which are common. There are reports of such payments being as much as 14 times higher than official fees [37] and that they are higher in northern provinces than in the south [9]. Other studies have also suggested that respondents to surveys factor in these unofficial payments when answering [5]. This would mean that "free health care" in the insurance scenarios would not be interpreted as free at all.

Another such factor is the risk that in reality, when using insurance, patients can risk longer waiting times and lower quality of care [31]. The scenarios, at least implicitly, assume the same quality in public health care for both insured and uninsured. These factors could explain a possible underestimation of WTP.

There are also reasons for why the true WTP might be relatively low, with quality of public health care being one. In comparison with private care, public care may, for example, be less accessible, have a smaller drug supply and meet patients with less respect [25]. Perhaps this could help to explain why our variable "hcneed" – if the household had been in need of health care during the last year – turned out to be a negative determinant of WTP. People with recent experiences of health care are better judges of what private as well as public care can offer.

Furthermore, in the methods section the potential importance of social capital was discussed. One part of this is the trust for the community that people have or don't have. If the respondents in our survey did not trust the local community to deliver what is specified in the scenarios, measurements of social capital – which we don't have – could have provided better insight into this problem. Another part of social capital is informal risk-sharing. Studies have shown that this is common in Vietnam, for example in the form of people borrowing money from relatives and friends to pay for health care, and that this may decrease the interest in health insurance [38].

## Conclusion

The goal for the Vietnamese government is to reach insurance or prepayment coverage for all citizens within a few years. Today, about half of the population is covered. Reaching the other half may prove to be harder than reaching the first. One way to study the possibilities for insurance expansion is to estimate the WTP for insurance – to find out how much other expenditure people are willing to sacrifice so that they can be insured or, put another way, what value they place on insurance.

This is, to our knowledge, the first such study in Vietnam. It has uncovered great scepticism of an insurance system; half of the respondents prefer an out-of-pocket system and the stated WTP is relatively low. It would, however, be wrong to conclude that it is too low. Under certain conditions, discussed above, people's WTP could sufficiently finance a health insurance system.

Our study leaves many questions for future research, some of which are: How much of the WTP result can be contributed to the product, public health care, and how much to competing informal risk-sharing networks? And how much can be contributed to the complexities of an insurance system in a setting where people are relatively inexperienced of such formal arrangements? It will take further quantitative and qualitative studies to uncover the answers to these questions.

Our findings on the determinants of WTP are, in this light, somewhat encouraging. WTP falls with increasing age and rises with more education. Older people may be less inclined to undergo change and therefore less ready to support a new, unknown system. People with higher education may be more confident in adjusting to, and trusting, a new system. These results are encouraging because they highlight a potential for public information schemes that could change the predominantly negative attitude towards health insurance that this study has uncovered. A key task for policy-makers is to win the trust of the population for a health insurance system, particularly among the old and those with relatively low education.

## Competing interests

The authors declare that they have no competing interests.

## Authors' contributions

CL performed the statistical analysis, drafted and revised the manuscript. NXT designed the questionnaire, was responsible for monitoring the interview process and was also responsible for drafting and revising the manuscript. NTKC, AE and LL participated in the conception, planning and design of the study and in the revisions of the manuscript. All co-authors read and approved the final manuscript.

## Appendix 1. Estimation of minimum health care costs for the section of the population in Bavi who prefer health insurance (compulsory or voluntary) to out-of-pocket health care payments

A little less than half of the respondents (48.4%) stated that they prefer either compulsory or voluntary health insurance over out-of-pocket payments for health care. We have estimated a minimum health care cost for this part of the population by using data from the Vietnam National Health Survey 2002 [39]. These data apply to the whole country so the following estimations in table 10 and table 11 is a rough approximation.

**Table A1:** Estimated number of enrolees and sickness episodes

Population in Bavi:	235,000
Percent of population insured:	17.4%
Population un-insured:	235,000*(1-0.174) = 194,110
Number expected to enrol in a health insurance scheme:	194,110*48.4% = 93,949
Number of sickness episodes per person per year*:	3
Number of sickness episodes among those enrolled in health insurance scheme:	93,949*3 = 281,848
Of which: episodes in inpatient care:	281,848*1.4%* = 3946
episodes in outpatient care:	281,848*28.1%* = 79,199
episodes of self-treatment:	281,848*65.9%* = 185,738
episodes of non-treatment:	281,848*4.6%* = 12,965

* Source: Vietnam National Health Survey 2002

**Table A2:** Estimated health care costs for the expected enrolees in a health insurance scheme in Bavi

	Utiliza- tion rates (1)*	Total sickness episodes (2)**	Sickness episodes (3)	Weight to distribute the private care episodes (4)	Private care episodes distributed to public facilities (5)	Expected sickness episodes in public facilities (6)	Health care cost per episode (7)*	Total costs 1000 VND (8)
Inpatients**		**3,946**						
CHC	0.158		623	0.173246	60	684	104,000	**71,097**
DHC	0.333		1,314	0.365132	127	1,441	242,000	**348,676**
Provincial and central	0.421		1,661	0.461623	160	1,822	242,000	**440,818**
Private and others	0.088		347					
Outpatients**		**79,199**						
CHC	0.32		25,344	0.659794	26,911	250,958	15,400	**3,864,753**
DHC	0.086		6,811	0.177320	7,232	14,044	43,800	**615,107**
Provincial and central	0.079		6,257	0.162887	6,644	12,900	43,800	**565,040**
Private and others	0.515		40,787					
Self-treatment**		**185,738**						
Non-treatment**		**12,965**						

**Total**		**281,848**				**281,848**		**5,905,492**

*Source: Ministry of Health and General Statistics Office: *Results of Vietnam National Household Survey 2001–02*. Hanoi; 2003.

Health care cost per sickness episode is health care cost per visit for outpatients or per admission for inpatients, including hospital fees, drugs, X-ray and laboratory tests. The cost does not include indirect (or non-medical) costs, such as costs for travelling, lodging and gifts.

**From table A1.

(3) = (1)*(2).

(4)CHC = (1)CHC/[(1)CHC + (1)DHC + (1)Provincial and central].

(4)DHC = (1)DHC/[(1)CHC + (1)DHC + (1)Provincial and central].

(4)Provincial and central = (1)Provincial and central/[(1)CHC + (1)DHC + (1)Provincial and central].

(5) = (4)*(3)Private and others.

(6)CHC = (3)CHC + (5)CHC + self treatment + non treatment.

(6)DHC = (3)DHC + (5)DHC.

(6)Provincial and central = (3)Provincial and central + (5)Provincial and central.

(8) = (6)*(7)

### Assumptions

Health care utilization patterns, health insurance coverage, number of sickness episodes per person and year and health care costs per episode in Bavi are similar to the national average.

All of the respondents who prefer health insurance over out-of-pocket payments in our study will choose to enrol in a health insurance scheme.

The length of stay at provincial and central levels is the same as district level. Because the hypothetical scheme allows for treatment at higher levels if needed, the insured will be compensated by a daily amount equal to the cost per bed day at district level. The cost for treatment at provincial and central levels is the same as the cost for treatment at the district level.

The non-treatment and self-treatment episodes will instead be episodes of out-patient care at commune health centres under the health insurance scheme.

Private health care users will use public health care with the same health care utilisation patterns as those of public health care users.

There are four major reasons why the costs in this system are lower than the current actual health care expenditure in the population. Firstly, none of the administrative costs for the insurance system are included. The costs should therefore be increased by 5–10%. Secondly, over half of the household health care expenditure in Bavi is spent on private health care. Thirdly, the household health care expenditure includes both direct (e.g. medical costs) and indirect costs (e.g. transportation cost). Finally, costs for care at the provincial and central levels as estimated in our hypothetical system are based on cost per bed day at the district level.
